# A multivariate grain size and orientation distribution function: derivation from electron backscatter diffraction data and applications

**DOI:** 10.1107/S1600576720014909

**Published:** 2021-02-01

**Authors:** Jesús Galán López, Leo A. I. Kestens

**Affiliations:** a MSE, Faculty 3mE, Delft University of Technology, Mekelweg 2, 2628 CD Delft, The Netherlands; b Department of Electromechanical, Systems and Metal Engineering, Faculty of Engineering and Architecture, Ghent University, Technologiepark Zwijnaarde 903, 9052 Ghent, Belgium

**Keywords:** grain size, texture, orientation distribution function, ODF, electron backscatter diffraction, EBSD, representative volume element, RVE

## Abstract

Two of the microstructural parameters most influential in the properties of polycrystalline materials are grain size and crystallographic texture. Although both properties have been extensively studied and there are a wide range of analysis tools available, they are generally considered independently, without taking into account the possible correlations between them. However, there are reasons to assume that grain size and orientation are correlated microstructural state variables, as they are the result of single microstructural formation mechanisms occurring during material processing. In this work, the grain size distribution and orientation distribution functions are combined in a single multivariate grain size orientation distribution function (GSODF). In addition to the derivation of the function, several examples of practical applications to low carbon steels are presented, in which it is shown how the GSODF can be used in the analysis of 2D and 3D electron backscatter diffraction data, as well as in the generation of representative volume elements for full-field models and as input in simulations using mean-field methods.

## Abstract 2

Grain size distribution and orientation distribution functions are combined in a single multivariate continuous grain size orientation distribution function (GSODF). Several examples of practical applications to low carbon steels are presented, in which it is shown how the GSODF can be used in the analysis of 2D and 3D electron backscatter diffraction data, and as input in full-field and mean-field crystal plasticity simulations.

## 1. Introduction   

When polycrystalline materials are studied, it is usually observed that local microstructural properties such as crystallographic orientation, grain size and aspect ratio are regularly distributed along the volume of the material with non-uniform frequencies. Parametric statistical distributions allow these frequencies, and therefore, in some way, the complexities of the microstructure, to be represented in an efficient manner. It is common in the materials science community to work with distributions of crystallographic orientations and grain sizes (Bunge, 1987 ▸; Randle & Engler, 2014 ▸; Ohser & Mücklich, 2000 ▸), since these two properties are some of the most influential in material behaviour at the macroscopic level. However, the possible correlation between them is usually overlooked, even though such a relationship can be expected in many – if not most – practical cases, since both properties directly influence, and are influenced by, the diverse metallurgical processes to which the material may have been subjected, such as heat treatments or mechanical deformation. When deformed mechanically, grains will respond differently depending on their size and orientation. During phase transformation, nucleation will occur according to variant selection rules and the new grains will grow with different velocities depending on their orientations and orientation relationships with neighbour grains. If the evolutions of orientations and grain sizes are intrinsically related, it is expected that their distributions will be too. The derivation and discussion of a multivariate grain size and orientation distribution function that takes into account this correlation is the main topic of this article.

### 1.1. Statistical description of microstructures   

As a result of the metallurgical processes that a material has undergone, certain orientations will be found with higher probability. It is then said that the material has developed a crystallographic texture. The main consequence of this texture is that the material will exhibit different macroscopic properties along different directions, *i.e.* anisotropic behaviour.

A distribution of crystallographic orientations can be represented either in a discrete form, when it consists of a simple list of orientations with their corresponding volume fractions, or using a continuous function, known as the orientation distribution function (ODF). The latter method offers the advantage of requiring a much lower quantity of data, which facilitates the numerical analysis of textures, assuming it is possible to find a function that properly fits the real distribution.

An efficient representation of a continuous ODF can be obtained by applying the harmonic series expansion method. This technique, developed by Bunge (1969 ▸, 2013 ▸) and later implemented by Van Houtte (1984 ▸, 1995 ▸) in the *MTM-FHM* software for texture analysis, consists of using a finite series of generalized harmonic functions such that the probability density of an orientation *g*, represented as a triplet of Euler angles 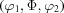, is given by 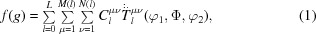
where 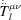 is a symmetrized generalized harmonic function and the factors 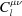 are called the *C* coefficients of the ODF, which can be considered as the set of independent parameters that define a texture. The expressions for the harmonic functions and the 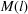 and 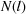 summation limits for different symmetries can be found in the book by Bunge (2013 ▸). This series will converge when its order (the value *L*) approaches infinity. In practice, the higher-order terms are usually small enough to be neglected. A value typically used in the analysis of textures with cubic symmetry is 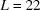, since this is the maximum suitable value when ODFs are extracted from pole figures obtained using conventional techniques (the suitable value is highly dependent on the texture maximum; for very strong texture, a value higher than 22 is required, but for conventionally processed metal products 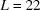 suffices).

In this work, only microstructures of cubic crystal symmetry will be considered, so this notation will be slightly simplified and the harmonic series expansion of an ODF will be represented as 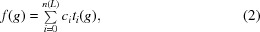
where for each possible combination of values of *l*, μ and ν a value of *i* corresponds such that 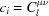, and 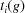 is the respective symmetrized generalized harmonic function. For the case of 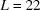, there will be a total of 186 *C* coefficients (of which the first one, 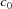, will always be 1). If orthorhombic sample symmetry is assumed, the number of independent coefficients for 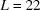 will be reduced to 125 (also including 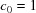).

Although the harmonic method was initially developed for the analysis of pole figures using X-ray diffraction techniques (Van Houtte, 1984 ▸), it is also commonly used nowadays for the study of measurements using the electron backscatter diffraction (EBSD) technique (Schwartz *et al.*, 2009 ▸); there are a large number of software packages available for the derivation of ODFs from experimental data and their analysis that employ this method or similar ones (Van Houtte, 1995 ▸; Bachmann *et al.*, 2010 ▸; Beausir & Fundenberger, 2017 ▸; Adams *et al.*, 1993 ▸). However, the harmonic series expansion method is not the only one available. There are also ODF calculation techniques based on the use of kernels different from the generalized harmonic functions (Matthies *et al.*, 1987 ▸; Helming & Eschner, 1990 ▸; Schaeben, 1994 ▸). In recent years, methods based on the use of the hyper-spherical harmonics have also been developed (Mason & Schuh, 2008 ▸). Although these methods present clear objective advantages over more conventional techniques from a mathematical point of view, their use is still rare in the field of texture analysis.

The study of crystallographic texture is often complemented with the study of the misorientations between different material points or grains. In its most complete form, the grain boundary network is described by the misorientation distribution function (MDF), which expresses the frequency of certain misorientations (Patala *et al.*, 2012 ▸). Usually, this information is further condensed in the form of a disorientation frequency function, which is limited to the distribution of disorientation angles, and does not include information about the rotation axis. Moreover, there have been several attempts to correlate misorientations and topological data (Beausir *et al.*, 2009 ▸; Adams *et al.*, 1987 ▸). In these studies, an MDF is calculated with respect to different distances, allowing one to subsume the ODF and MDF and include also indirect information about grain sizes and shapes, and their arrangement in the microstructure.

Another property that directly influences macroscopic behaviour is grain size. Mechanical properties, for instance, are influenced by the Hall–Petch effect (Hall, 1951 ▸; Petch, 1953 ▸), which accounts for the higher mechanical strength of materials with smaller grains due to the difficulty of the dislocations overcoming grain boundaries. In general (although not always), grain sizes are distributed such that the frequency of ratios with respect to an average size 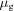 can be approximated by a normal distribution with standard deviation 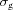 on a logarithmic scale. In this case, the grain sizes will approximately follow a log-normal distribution (Bergmann & Bill, 2008 ▸): 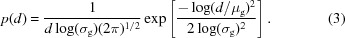
Here, *d* is the grain size, and the 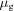 and 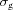 values are defined as the geometric average and geometric standard deviation of the grain sizes, respectively, both of them weighted with respect to volume: 
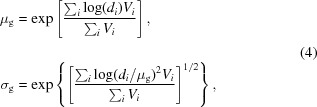
with 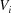 the volume of grain *i* and 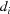 its equivalent diameter such that 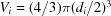.

### 1.2. Combining grain size and orientation distributions in a single multivariate distribution function   

As previously said, it cannot be expected that the distributions of grain size and crystallographic orientation will be independent. Therefore, they will be better represented using a joint probability function. From a theoretical point of view, combining the distributions of grain orientations and sizes in a single multivariate function should enable a more accurate representation of the real microstructure. Unlike a traditional ODF, a combined distribution would allow one to distinguish the cases in which an orientation with a large volume fraction is the consequence of a relatively low number of large grains from those where there are a large number of small grains. This can make a big difference, for example, when creating a virtual representation of a microstructure for the simulation of a deformation process. A combined distribution would also be better suited to the analysis of experimental data for the study of processes related to the growth of grains, such as phase transformation, recrystallization and grain growth phenomena, since it would allow identification of correlations that cannot be observed using independent distributions. A similar argument can be made with respect to misorientations. If it is expected that grain sizes and crystallographic orientations will be correlated, this correlation should also manifest when the relationship between grain sizes is taken into account when calculating the distribution of misorientations.

In this article, a simple method to derive a multivariate grain size and orientation distribution function, or GSODF, in the form of a continuous function is presented. The expression of the joint distribution as a single continuous function allows the information to be condensed in a simple analytical expression which facilitates further analysis. In Section 2, a precise formulation for this function is given. Section 3 shows several examples of how the GSODF and grain-size-dependent misorientations can be applied to the analysis and simulation of low carbon steel microstructures. Finally, Sections 4 and 5 discuss the obtained results and present some conclusions.

## 2. Grain size and orientation distribution function   

The GSODF is defined as a multivariate function 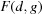 of grain size *d* and crystallographic orientation *g*. If the ODF corresponding to the grains of size *d* is given by the function 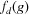 and the grain size distribution of the material is given by a function 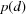, then the GSODF is simply given by the product of *p* and 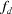: 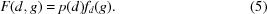


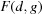
 can be interpreted as the joint probability density function of finding a material point with crystallographic orientation *g* that belongs to a grain with equivalent diameter *d*. The conventional ODF and grain size frequency function can be derived from the GSODF as the marginal distributions of *g* and *d*, respectively.

In the following, a method is presented to derive the above function from experimental data or virtual microstructures.

### 2.1. Grain definition and grain size distribution   

The starting point for the method is a list of grains with the size and crystallographic orientation of each of them.^1^ Such a list can easily be obtained from EBSD experimental data using conventional analysis techniques. For example, a simple pipeline could consist of some basic cleaning of the raw data (removing or replacing low-quality points), the definition of grain boundaries on the basis of a misorientation limit (usually between 5 and 10°) and the calculation of grain orientations, averaging over their points. Grain sizes are obtained from the number of data points corresponding to each grain and the step size employed in the measurements. Virtual microstructures can be processed in a similar manner.

From this list of grains, it is trivial to extract the grain size distribution and fit to it a probability function. The most suitable function will depend on the specific microstructure under consideration. In many practical cases, a log-normal distribution is considered a reasonably accurate approximation. However, the method presented here would also work for any other grain size distribution function [see, for example, Vittorietti *et al.* (2019 ▸)], and even for discrete distributions.

### 2.2. ODF by grain size   

The whole list of grains is subdivided into *N* bins corresponding to different size ranges, such that the grains in bin *n* have a size in the range 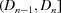 (with 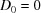). The range limits 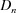 can be defined on the basis of different strategies, for instance to get bins of the same width, or such that the number of grains in each bin is the same. To each bin corresponds an equivalent diameter 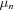, given by the geometric average formula in (4) applied to the grains in the bin (note that this value will be, in general, different from the middle point between 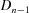 and 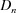).

An ODF analogous to (2) is then calculated for the subset of grains in each of the bins using the series expansion method described in Section 1.1. It is assumed that this ODF represents the distribution of orientations for grains of the corresponding equivalent size, 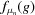. Since the harmonic functions 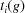 are always the same, any variation on the obtained ODFs will be the result of a variation of the *C* coefficients. Therefore, the *C* coefficients must be dependent on the grain size [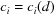]: 
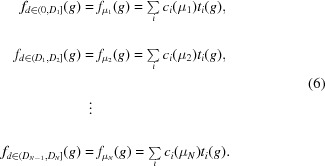



The set of 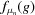 functions define a discrete set of ODFs for a fixed number of grain sizes. In order to express the GSODF as a continuous function, it is necessary to express the dependence of the *C* coefficients on grain size as a series of continuous functions 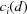, such that the GSODF can be evaluated for any grain size. These functions may be difficult to find for some or all values of *i*, depending on the particular microstructure under study. Nevertheless, if the function is not trivial to find, it can always be approximated by linear interpolation between the coefficients obtained for each size. As an extreme case, a first-order approximation of the real function may be obtained by dividing the initial set of grains into only two bins. In the particular case a linear relationship is assumed, the grain-size-dependent ODF will be given by 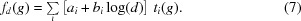
An ODF linearly dependent on 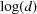 is therefore characterized by a set of intercept coefficients 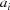 and a set of slope coefficients 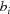. The function is linearized with respect to 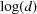, instead of *d*, in accordance with the use of geometric averages to calculate equivalent sizes.

In many problems, it can be convenient to have a straightforward way to calculate the ODF for the equivalent grain size (either of a whole microstructure, or for the small range in which it is used for interpolation if that is the case). Therefore, it can be more practical to present the grain-size-dependent ODF in the alternative form 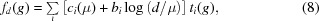
where μ is the geometric average of the diameters of all the grains in the range for which the linear relationship is considered valid.

### 2.3. GSODF   

Once the *C* coefficients are expressed as functions of grain size, all that is needed to obtain the GSODF is to multiply the size-dependent ODF by the grain size frequency function as in (5): 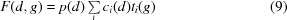
and for the linear case 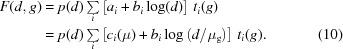
This expression condenses, using only two sets of *C* coefficients, the information contained in the original list of grains, and additionally allows the calculation of probabilities for orientation and grain size values not present in the original data. The set of 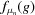 ODFs in (6) can be considered as an intermediate representation between the continuous GSODF and a discrete list of grains, in which probabilities for different orientations are described as continuous functions, but only for a discrete set of grain sizes.

### 2.4. Disorientation and size ratio joint distribution   

For the study of misorientations, the disorientation distribution is calculated with respect to size ratios. If, for each grain *i*, the boundaries are identified such that the disorientation 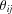 and volume ratio 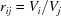 are known, then the probability density function will simply be given by the boundary area between all the grains with a certain size ratio and disorientation angle with respect to the total boundary area: 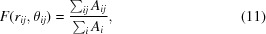
where the convention is that the area between two non-neighbour grains is zero. The same formula can be applied to 2D microstructures, simply replacing volumes by areas and boundary areas by boundary lengths.

In the next section, the validity of the assumptions made is tested for a number of particular cases. The goal is to study the capacities of the GSODF function to describe real microstructures and show several examples of its usage.

## 3. Applications   

The method presented in the previous section has been applied to several cases related to the analysis and modelling of low carbon strip steel microstructures. Texture development and grain size in these materials are of the utmost importance because, although rolled steels always show similar characteristics, subtle details resulting from the processing route followed can lead to important differences in the final properties of the material (Kestens & Pirgazi, 2016 ▸).

The first example demonstrates how the GSODF can be obtained from 3D EBSD experimental data for an extra low carbon (ELC) steel. Additionally, the GSODF is calculated from a single EBSD layer in order to evaluate the method when only 2D EBSD data are available. Further examples are presented corresponding to two different grades of dual phase (DP) steel, each of them characterized using 3D EBSD, and to two interstitial free (IF) steels after warm and cold rolling. The goal of these examples is twofold: first, they allow observation of the grain size dependence of texture in a wider range of microstructures; and second, they serve to illustrate several uses of the GSODF and related concepts. It is also shown how an obtained GSODF can be applied in modelling problems.

### 3.1. Materials and methods   

**Figure 1:**
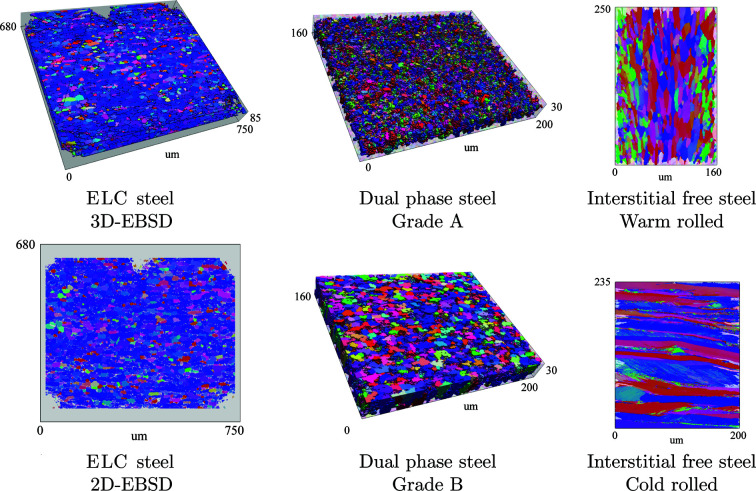
Experimental EBSD data. Non-valid points (small grains located on the edge) are shown semi-transparently. The size of the samples is indicated in the figure (all dimensions are in µm). Further details are given in Table 1 ▸.

An overview of the EBSD experimental data available for each of the materials is presented in Table 1 ▸ and Fig. 1 ▸. The table shows the number of processed points and the resulting number of total and valid grains, as well as the layer spacing in 3D EBSD. In the figure, the grains considered for the calculation of the GSODF of each material are displayed.

#### 3.1.1. Experimental procedure   

3D EBSD measurements were carried out using the sequential sectioning technique via automated mechanical polishing. In this technique, consecutive steps of sample preparation and EBSD scanning are employed to obtain 2D EBSD sections which are later used for the reconstruction of the 3D microstructure. A field emission gun scanning electron microscope (QUANTA450 FEI) equipped with an EDAX-TSL EBSD system was employed to scan the 2D sections, and an automated polishing machine was used to perform the parallel sectioning with uniform rotational speed. Plane parallelism of the individual layers is also controlled by a series of micro-Vickers indentations around the measured area. The final 3D microstructure was obtained by processing the collected scans with the alignment method of Pirgazi (Pirgazi *et al.*, 2015 ▸; Pirgazi, 2019 ▸).

Conventional EBSD was performed in the cold-rolled IF steel using a JEOL JSM 6500F field emission gun scanning electron microscope with an EDAX/TSL detector. For the warm-rolled one, an HKL Nordlys II detector was used, also attached to a JEOL JSM-6500F field emission gun scanning electron microscope.

#### 3.1.2. Data processing and basic characterization   

The *Dream3D* software (Groeber & Jackson, 2014 ▸) was used to process the EBSD data and to obtain the list of grains with crystallographic orientation and volume described in Section 2.1. Grain boundaries are defined with a misorientation of 5°, with the exception of the rolled IF steel samples for which 2° is used, in order to capture the high number of subgrains in the samples. Bad data points are removed and substituted by accepted neighbouring points (allowing a minimum defect size of 5 points), and average rotations and volumes are calculated for each grain. Other additional quantities not used in the GSODF calculation, such as aspect ratios and misorientations, are also calculated at this stage. The small grains on the edges of the sample are declared non-valid with the objective of avoiding the bias introduced by sectioned grains. As a result of this filtering, the number of valid grains shown in Table 1 ▸ is obtained.

**Figure 2:**
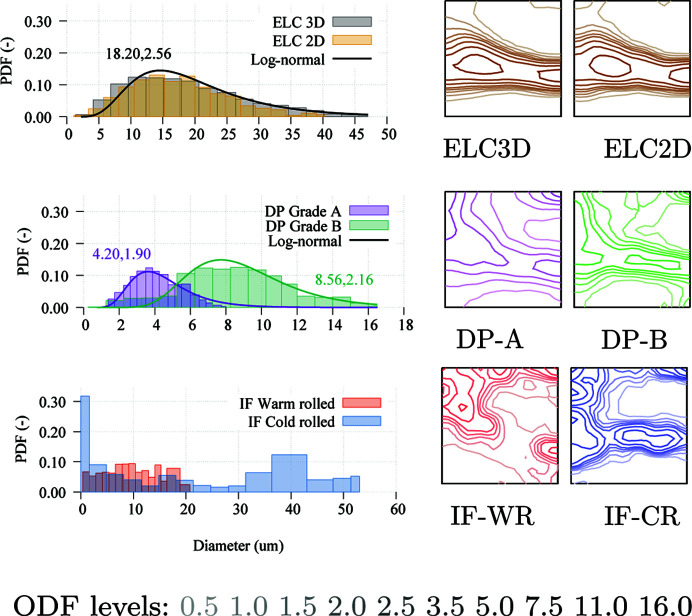
Grain size distributions and ODFs for the ELC, DP and IF steels. For the ELC and DP grades, the grain size distribution graph also includes the fitted log-normal (as a solid line) and the corresponding 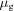 and 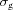 values. Only the 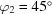 section of the ODF is displayed, in a different colour for each material (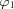 grows from 0 on the left to 90° on the right and Φ from 0 at the top to 90° at the bottom). Below, the intensity levels of the ODFs in random units.

Once the grain list is available, and before proceeding with the fitting of the GSODF function, a more traditional characterization of the material is performed. Fig. 2 ▸ shows the grain size distributions and the φ = 45° sections of the ODFs calculated for each of the cases presented in Fig. 1 ▸. For the ELC and DP materials, a log-normal function was fitted to the grain size distribution (the corresponding geometric average and standard deviation values are given in the figure too). ODFs were calculated using the *MTM-FHM* software (Van Houtte, 1995 ▸), using a Gaussian spread of 5° and 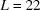 with triclinic sample symmetry (however, for simplicity, only the values for 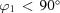 are displayed in the figures). Important fibre and individual components for cubic crystals are described in detail by Kestens & Pirgazi (2016 ▸).

### 3.2. GSODF of ELC steel: fitting from 3D and 2D EBSD data   

The first example shows in detail how the GSODF can be fitted from 3D and 2D EBSD data. The ELC steel experiment described in the previous section is used with this purpose, taking advantage of the extensive data set available, consisting of more than 28 000 valid grains.

The total set of grains is divided into bins by size ranges, such that all bins have an equal number of grains, and the ODF for each of these bins is calculated using the *MTM-FHM* software. A fixed number of grains per bin is chosen instead of using other binning strategies because this way the ODFs of all bins will be calculated in similar conditions. If, for instance, bins of the same width were used, the ODF of each bin would be calculated with a different number of grains, and the calculated ODF would appear artificially sharper for those bins with a low number of grains. The number of bins, five, is arbitrarily chosen; the number of bins will be further discussed in Section 4.

**Figure 3:**
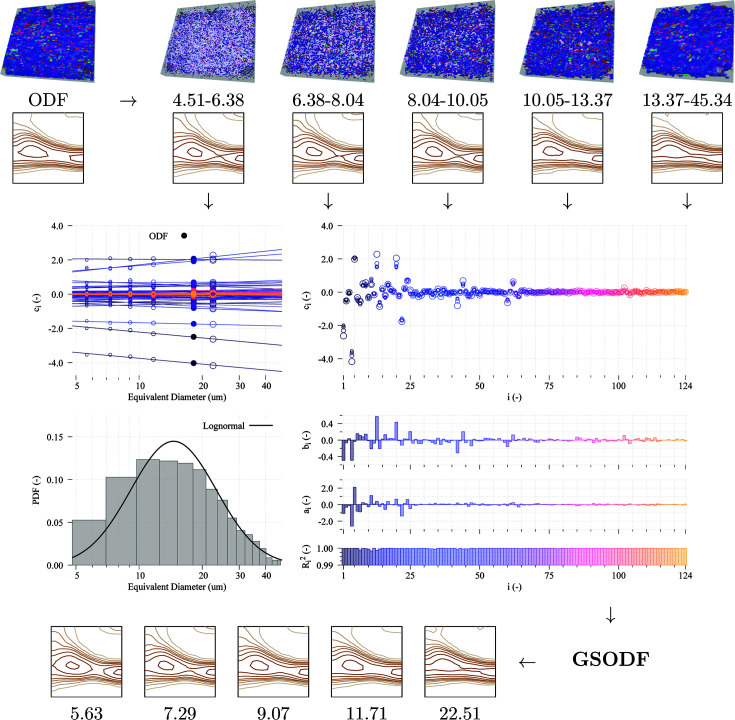
GSODF fitting for ELC material using 3D EBSD data. At the top, the grains selected for each of the bins and the corresponding size range (in µm) are displayed. The graphs in the middle show (from top left going clockwise) the dependence of the *C* coefficients on grain size (using a different colour for each coefficient), all the *C* coefficients (in order) at the top right, the fitted 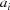 and 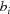 coefficients, and the grain size distribution (in logarithmic scale). Below, the plots of the ODFs predicted from the fitted GSODF for the equivalent sizes corresponding to each of the bins at the top are shown. ODF levels are the same as in Fig. 2 ▸.

Fig. 3 ▸ shows, at the top, how the total set of grains is divided into bins and the 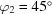 section of the ODFs calculated for each of them. All the obtained textures are quite similar, although a small increment in sharpness is observed as the grain size increases. While the intensities of the predominant gamma fibre components grow, those of the rotated cube components decrease. Another difference is that the intensities along the gamma fibre are more equally distributed when the grain size increases, whereas for small grains there is a distinct peak in the 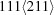 component.

The figure also includes a graph of the obtained *C* coefficients with respect to the logarithm of the equivalent diameter. As can be seen in the graph, in this case the behaviour of the *C* coefficients is actually very close to linear for the entire size range, so formula (9) can simply be replaced with (10). The values of the intercept and slope coefficients are then obtained by applying the least-squares method for each of the coefficients. The fitted lines are shown in the graph too, as well as the 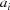 and 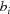 coefficients and the 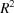 values obtained in each of the fittings. A remarkably good fit is obtained: all the 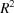 values are close to 0.999 and the *C* coefficients of the measured ODF in Fig. 2 ▸ (displayed with filled circles in the graph) lie on the fitted line.

Finding such a strong linear relationship was not an expected result. The result is welcomed because it greatly simplifies the calculations, but it also raises many questions such as what might be the underlying reason for this linear dependence; will this behaviour be observed in more materials with a wide variety of microstructures, or is it just a coincidence? These issues will be further discussed in Section 4.

Finally, as an additional validation, the ODFs for the equivalent sizes of each of the bins calculated using the obtained GSODF, shown at the bottom of Fig. 3 ▸, can be compared with the size-dependent ODFs previously calculated (at the top of the figure). The predicted and measured textures are almost indistinguishable.

**Figure 4:**
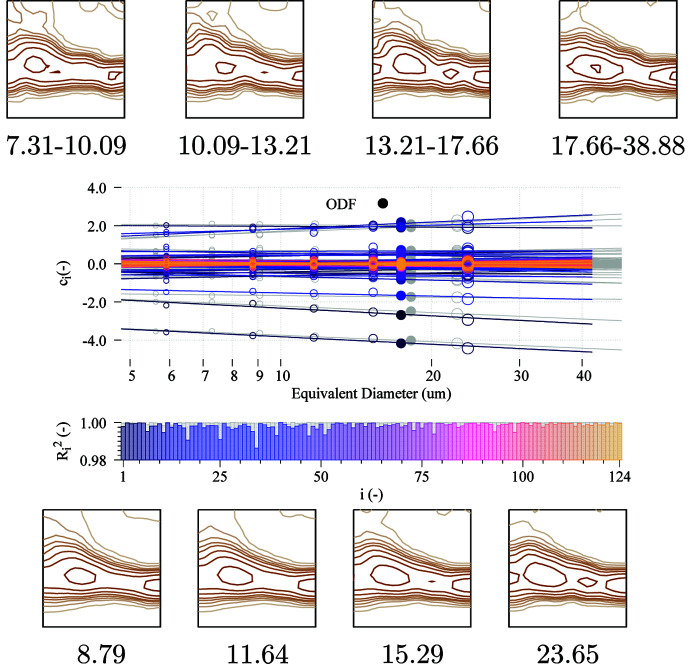
GSODF fitting for ELC material using a single 2D EBSD scan. Grain-size-dependent ODF plots for bins of different size ranges are shown at the top (*cf*. global ODF plot in Fig. 2 ▸), and predicted ODFs at the bottom. Sizes are given in µm. The middle graph shows the *C* coefficients and the fitted lines (results from the 3D EBSD are shown in grey for comparison). The 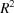 values from the fitting are also displayed. ODF levels are the same as in previous figures.

The results obtained from the 2D experiment are shown in Fig. 4 ▸. In this case, the fitting process is performed using only the 2D EBSD data of the middle layer in the 3D EBSD experiment. Other layers are not used either in the preliminary data processing or for the GSODF fitting. As in the 3D case, the set of grains is first divided into five bins; then the corresponding ODFs are calculated and, after fitting of the GSODF, used to predict the ODF for the equivalent size of each bin.

The number of grains available in a single 2D scan is much lower than the total number in the 3D data set (2659 instead of 28 605, so the ODFs of the bins are calculated using approximately 530 grains). Hence, the calculated ODFs are not so smooth as when using the 3D data. As a result, the quality of the fits of the 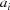 and 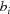 coefficients is slightly lower too, although the obtained 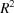 values are still higher than 0.99. Since the GSODF combines the data of all the bins’ ODFs, the ODFs predicted for the equivalent sizes are closer to the global ODF calculated from the 2D EBSD shown in Fig. 2 ▸ than to the ODFs of the bins. They are also very close to the ones obtained from 3D EBSD.

### 3.3. Comparison of two different DP grades   

Additionally, the GSODF is used for the analysis of the two DP steel grades. The goal is to observe the utility of the GSODF to compare materials which, although different, are relatively similar, and determine if the GSODF can be used to differentiate between them. DP steels are chosen as a characteristic example of high-strength steels widely used in the automotive industry (Galán *et al.*, 2012 ▸). The DP-A material is a DP steel developed in the laboratory for research purposes, while DP-B is a typical commercial grade. Commonly, there is between 10 and 15% volume fraction of martensite in these materials. Identifying the phases is further complicated by the deformation present in the material and the method used to recreate the 3D microstructure from 2D measurements. In order to avoid these complications, the GSODF is calculated for the combined ferrite and martensite phases, after points with a very low image quality are removed. As Fig. 2 ▸ shows, the resulting grain size distribution closely resembles a log-normal, although the DP-B distribution could in fact be a binormal distribution, resulting from the combination of ferrite and martensite distributions. The fitting is performed using 3D EBSD experiments following the same procedure as described in the previous section, with the only difference being that eight different size bins are used. Size ranges are defined again such that all bins have the same number of grains.

**Figure 5:**
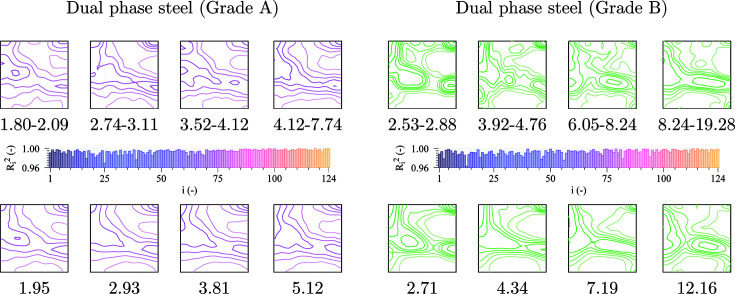
GSODF fitting for DP-A and DP-B steel grades. The figure includes the φ = 45° sections of the ODFs calculated from several size bins at the top (*cf*. global ODF plots in Fig. 2 ▸), the 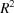 values obtained by fitting the 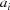 and 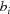 coefficients in the middle, and plots of the predicted ODFs at the bottom (ODF levels: see Fig. 2 ▸).

Fig. 5 ▸ shows the size-dependent experimental textures and the ones predicted by the GSODF for the corresponding equivalent sizes (in order to keep the figures simple, ODF sections are displayed only for some of the bins), as well as the 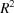 values from the fitting. Also, in the present case, the *C* coefficients exhibit a strong linear dependence on 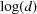. The textures for all sizes in grade A are quite similar, and therefore this material has a low texture dependence with respect to grain size. The GSODF can easily reproduce this result. However, in the DP grade B, which has much larger grains, there is a clear dependence of the intensity of the alpha and gamma fibre components on grain size. Although there are some problems reproducing the high intensities observed at lower grain sizes, in general the fitted GSODF satisfactorily reproduces the experimental textures.

**Figure 6:**
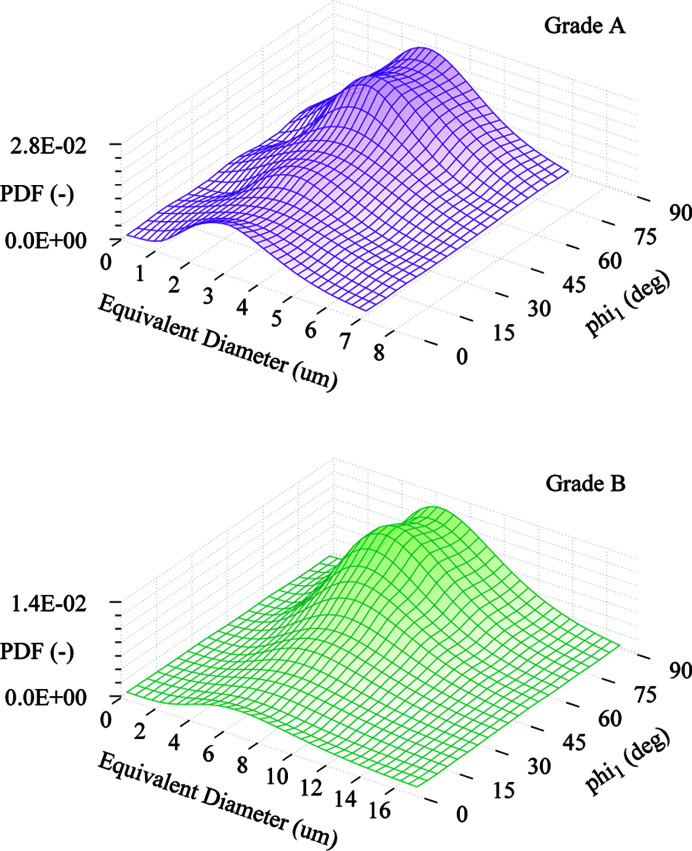
Distribution of grains with crystallographic orientation in the gamma fibre by size for the DP grades A and B. Frequency is given in random units. (Note, the scale of the ‘Equivalent Diameter’ axis is two times larger in the top graph.)

Once the GSODF has been calculated, it can be used to estimate the frequency of any combination of grain size and crystallographic orientation. In the case of DP and other high-strength steel products, some of the most interesting texture components are those in the gamma fibre, due to their importance for the forming behaviour (Galán-López & Kestens, 2018 ▸). Fig. 6 ▸ shows the intensity corresponding to each of the gamma fibre components represented with respect to grain size in a 3D graph. It is shown here as an example of how the GSODF can be used to evaluate and compare sheet steels. For instance, the graphs clearly show the average size of the grains in the gamma fibre, and that the distribution of orientations along the fibre is more uniform in grade A. Both observations can be directly linked with forming behaviour: a smaller size will mean more work is necessary to deform the material, while a more homogeneous gamma fibre will be translated into lower planar anisotropy. Equally important is the observation that high gamma fibre intensity corresponds to lower-than-average grain size, which implies that good deep drawability and high strength can go hand in hand. This information can also be applied in the study of the formation mechanism of gamma fibre grains.

### 3.4. Influence of rolling conditions on microstructural development of IF steel   

The last materials studied are the two IF steel grades rolled under different conditions. While one sample was subjected to a thickness reduction of 77.5% in a traditional cold-rolling setup, the other one was processed using asymmetric rolling up to a thickness reduction of 80%, with reheating up to 1173 K between rolling passes. It is well known that warm- and cold-rolled microstructures exhibit different recrystallization behaviour (Yoshinaga *et al.*, 1999 ▸), so there must be some differences in the microstructures formed during rolling. The goal of this example is to discuss the potential of the GSODF to study this problem and, more generally, for the analysis of highly deformed microstructures.

3D EBSD data are not used in this case, only common 2D scans of a relatively small size (see Table 1 ▸ and Fig. 1 ▸). The rolled sheets were not subsequently annealed in order to preserve the subgrains in the deformed microstructure. Grain boundaries were defined with a misorientation of only 2° (different from the 5° used in previous sections) to include these subgrains in the analysis. As a result, the grain size distribution is highly biased towards the smallest grain sizes and does not match a log-normal. Therefore, the GSODF will be based on a discrete grain size distribution.

**Figure 7:**
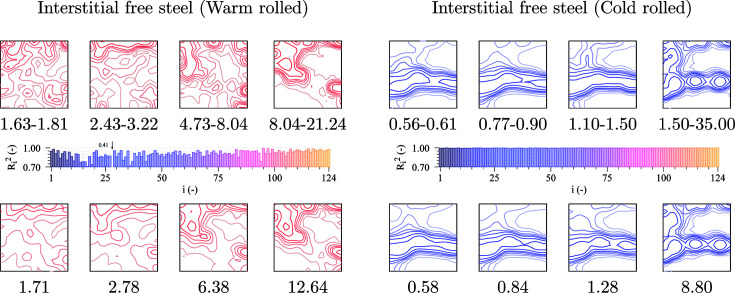
GSODF fitting for IF warm- and cold-rolled steels. The figure includes the φ = 45° sections of the ODFs calculated from several size bins at the top (*cf*. global ODF plots in Fig. 2 ▸), the 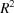 values obtained by fitting the 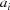 and 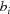 coefficients in the middle, and predicted ODFs at the bottom (ODF levels: see Fig. 2 ▸).

Fig. 7 ▸ shows the obtained results, in the same format as Fig. 5 ▸. The fitting is performed using eight bins with the same number of grains. It is observed that the fitting quality is lower than in previous cases, especially for the warm-rolled material, as could be expected from the low number of grains. Nevertheless, the fitted GSODF can still predict the dependence of the ODF on grain size with remarkable accuracy.

It is interesting to observe that the variation of the ODFs with grain size is quite different for the two materials. For the cold-rolled steel, a strong gamma fibre component is observed for all grain sizes, but only the largest grains exhibit a comparatively strong alpha fibre component (these strong alpha fibre components can also be clearly observed in the global ODF presented in Fig. 2 ▸). The ODFs obtained for the warm-rolled material for small and large grains are completely different, with the particularity of a strong peak in the component 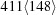 for the largest grains, which is also one of the dominant components in the global ODF of Fig. 2 ▸. Although finding the reason for this behaviour is out of the scope of this article, this result presents a clear example of the kind of new insight that the GSODF offers for the solution of this sort of problem.

In order to show another example of how studying the correlation between crystallographic orientations and grain size can aid in the investigation of microstructural data, the analysis is complemented with the study of the grain-size-dependent distribution of misorientation angles. Although misorientation data cannot be extracted from a simple list of grains as used until now, this information can be easily retrieved from the original EBSD data (or from a virtual microstructure). Using *Dream3D*, the neighbours of each grain are found, as well as the area of the common boundaries between them. This information is then used to calculate the misorientation distribution.

The misorientation data available on a grain-by-grain basis can be processed by bins, analogously to how it is done for the calculation of the ODFs, in order to calculate the disorientation and size ratio distribution function discussed in Section 2.4 which considers, for each boundary, not only the disorientation angle between the grains at each side of the boundary but also their relative sizes. The additional information in this multivariate distribution enables a more complete description of the character of the grain boundaries. More precisely, it allows determination of the preferential surroundings of grains, where grains are characterized by a size class and a crystallographic orientation.

**Figure 8:**
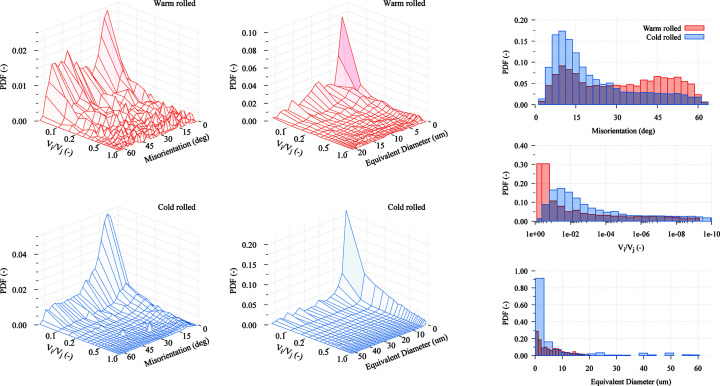
Distribution of misorientation angles and size ratios at boundaries. For each material is shown: area frequency of boundaries between grains with certain misorientation angle and volume ratio in the left 3D graph, area frequency of boundaries between grains with certain volume ratio with respect to size in the right 3D graph, and aggregate distributions with respect to boundary area of misorientation angles, volume ratio and size, on the right.

Using this method, the graphs corresponding to different distributions for the IF steels were calculated, and they are shown in Fig. 8 ▸. The 3D graphs in the figure represent the probability density function for boundaries between two grains with a specific volume ratio and misorientation angle, and also the distribution of volume ratios with respect to grain size. The difference between the two microstructures is clearly seen in the graphs: while most interfaces in the cold-rolled material are low-angle boundaries between large and small grains, the distribution obtained for warm-rolled material is much more spread out, with boundaries of different size ratios and misorientation angles. The figure also includes the marginal distributions for misorientation angle (the conventional misorientation distribution), volume ratio and grain size, with respect to boundary area.

The obtained graphs show some non-obvious facts about the grain boundaries in the material. Normally, only misorientation distributions are used (graph at the top right of the figure). It is also interesting to observe the large portion of these boundaries which belong to very small grains, especially in the cold-rolled material (bottom-right graph). However, when looking at the distribution of size ratios (middle-right graph), it is seen that most of the boundaries are between grains of different size. The 3D graphs in the centre summarize this result. The conclusion that can be drawn when also taking misorientations into account (left 3D graphs) is that there are a very large number of small grains – with a large boundary surface – surrounding larger grains, while the boundary area between grains of the same size is very low. This observation suggests that, during cold-rolling, these smaller grains form from the fragmentation of larger grains, contradicting the commonly accepted view in several fragmentation models where it is assumed that grains are divided into units of similar size. It is also remarkable that such a conclusion cannot be easily derived from the simple observation of EBSD maps (*cf.* Fig. 1 ▸), where the relative area of small grain boundaries with respect to that of large grains is easily overlooked.

### 3.5. Representative volume element generation   

The increasing usage of spatially resolved models that require full topological information, both in phase transformation simulations using phase-field methods or cellular automaton models and in mechanical problems using crystal plasticity finite element simulation or crystal plasticity fast Fourier transform, makes it necessary to generate accurate representative volume elements (RVEs) (Groeber & Jackson, 2014 ▸; Bargmann *et al.*, 2018 ▸; Kraska *et al.*, 2009 ▸; Roters *et al.*, 2019 ▸). A compromise must be made between the computational resources available and the size of this RVE, and therefore its capacity to represent the properties of the material with statistical validity. The GSODF allows one to produce more representative virtual microstructures by correlating orientation and size properties in the generated RVEs.

As an example, the ELC steel microstructure analysed in Section 3.2 is reproduced using RVEs generated with *Dream3D*. Three synthetic microstructures of different sizes are created with the log-normal grain size frequency function shown in Fig. 2 ▸. Both the number of cells and the size of these cells change from one RVE to another. The dimensions and resolution, as well as the total number of grains in the generated microstructure, are displayed in Table 2 ▸. The goal of using different sizes and resolutions is to offer an overview of the capacities of the GSODF when employed for the generation of RVEs to be used in a wide range of full-field simulations, which may have different requirements.

Crystallographic orientations are assigned to the grains, first dividing them into five bins of different size ranges. As is the case for the fitting of the GSODF from experimental data, size ranges are chosen so that all bins have the same number of grains. Then the ODFs for the equivalent size of each bin are calculated from the GSODF previously found in Section 3.2, and a discrete texture is generated using the *MTM-FHM* software. Finally, the orientations in this discrete texture are randomly assigned to the grains in the corresponding bin.

**Figure 9:**
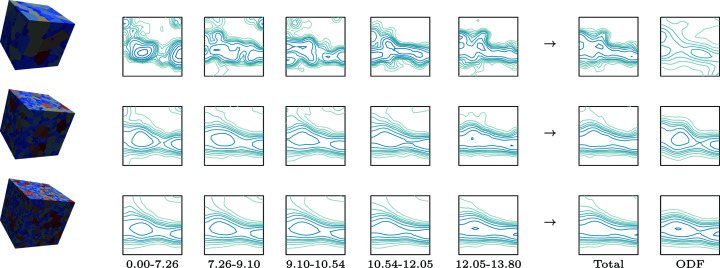
The three RVEs generated with the sizes given in Table 2 ▸. The virtual microstructures produced by *Dream3D* are shown on the left (colours indicate grain size: from blue for small to red for large). On the right, 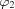 sections of the ODFs calculated after assigning orientations by grain size, and the global ODFs of the whole RVE generated using, respectively, the GSODF and the conventional ODF (levels: see Fig. 2 ▸). See also Table 2 ▸.

Fig. 9 ▸ shows the three RVEs generated and the corresponding ODFs calculated for each size bin, as well as the global ODF obtained when aggregating all the grains. The smallest RVE does a poor job reproducing the experimental texture. This result could be expected, since the number of grains is very small: the microstructure consists of less than 250 grains, which means that there will be less than 50 grains in each of the bins, a very low number for the calculation of a smooth ODF. On the other hand, the two largest RVEs successfully represent the material texture and its dependence on grain size.

The figure also includes plots of the ODFs corresponding to RVEs with the same topology to which orientations were assigned, by simple random sampling, from the ODF in Fig. 2 ▸. Not only does the GSODF allow us to express correlations that are neglected when using a conventional ODF, but the use of the correlation with grain size also allows us to better reproduce the global ODF without requiring complex orientation assignment schemes.

### 3.6. Mean-field modelling   

As opposed to full-field models, which employ virtual microstructures in the form of RVEs, mean-field models allow the solution of macroscopic problems without finding a detailed spatial solution. Instead, these models rely on homogenization methods to find an ‘average’ solution based on a non-topological (*i.e.* statistical) description of the microstructure. Although the output produced by mean-field models is much more limited, they are extremely useful for the calculation of macroscopic behaviour, since they can usually take into account a much larger number of grains with a fraction of the computing time and pre-processing work needed to perform full-field simulations. Mean-field models can also benefit from the use of the GSODF.

The viscoplastic self-consistent (VPSC) model of Lebensohn & Tomé (1993 ▸) is used in this section to demonstrate how the GSODF can be applied to simulations using a homogenization model. The VPSC model predicts the mechanical behaviour of multiphase polycrystalline materials under the assumption that grains behave as ellipsoidal inclusions in an homogeneous matrix with the aggregate properties of the polycrystal. The microstructural data considered in the model are the volumetric fraction of each phase, the average grain shape (either by phase or by crystal orientation) and a discrete crystallographic texture for each phase. Additionally, the model uses a number of input parameters related to the properties of each phase, such as crystal structure, slip systems and hardening parameters. Further details about the model are given by Lebensohn & Tomé (1993 ▸), Galan Lopez (2014 ▸) and the VPSC manual (Tomé & Lebensohn, 2009 ▸).

The *VPSC90* implementation (Galán *et al.*, 2014 ▸) is used to perform simulations of a deformation process under uniaxial tension conditions. Microstructural input data are taken from the ELC 3D EBSD experiment described in Section 3.2. The capability of the model to work with multiphase materials is exploited here to simulate a microstructure with different grain sizes. A total of eight different phases, corresponding to eight grain size classes, are considered. The volume fraction of each grain size class is taken from the frequency given by the grain size distribution (Fig. 2 ▸). For each size, an ODF is calculated from the GSODF previously fitted, and the *MTM-FHM* software is used to extract a discrete texture of 500 grains (the model will consider each size class proportionally to the given fraction, so the number of grains can be freely chosen). The properties of all phases are defined alike, except that hardening parameters are modified according to the Hall–Petch relationship and different grain shapes are defined depending on size. These shapes are extracted from the *Dream3D* software, which allows post-processing of the 3D EBSD data set by attributing an equivalent ellipsoid to each grain and then considering the geometric average for the grains in each size bin.

It is considered that deformation will be accommodated by slip on 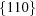 and 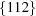 planes along 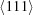 directions. The hardening behaviour for all slip systems is defined according to a modified Voce law (Tomé *et al.*, 1984 ▸): 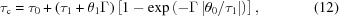
where Γ is the shear strain accumulated in the family of slip planes with critical shear stress 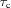. The 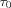, 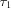, 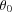 and 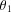 parameters are estimated from typical values for low carbon steel (Smith *et al.*, 2006 ▸), such that the law responds to the Hall–Petch relationship: 
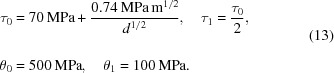
For comparison, an additional simulation is performed in which the material is modelled using a single phase. The texture for this phase is calculated using the global ODF (Fig. 2 ▸), hardening parameters are calculated by substituting the average grain size in (13), and the shape is defined as the (geometric) average over all the grains.

**Figure 10:**
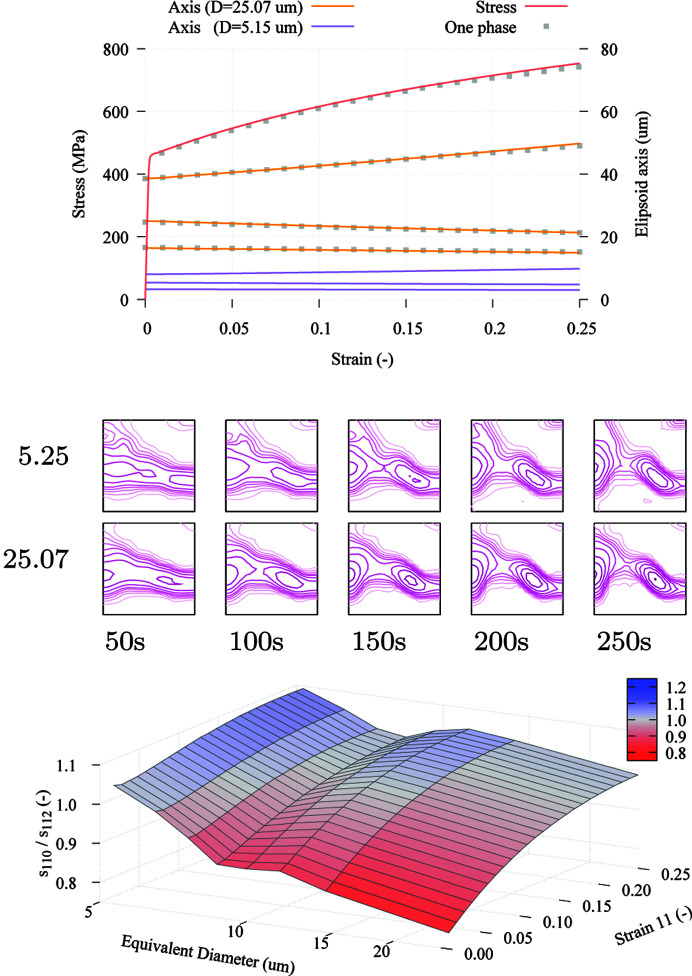
Results of VPSC simulation. Top: tensile curve and evolution of equivalent ellipsoid axes with respect to strain. Middle: simulated texture evolution for the smallest and largest grains. Bottom: evolution of the ratio of slip system activity in the 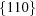 and 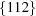 planes during deformation represented with respect to grain size.

Fig. 10 ▸ shows several examples of the output that is produced by the model. In the displayed tensile diagrams (with points and solid line), results from the two simulations are compared. A small difference is observed in the obtained curves. The graph also includes the evolution of the equivalent ellipsoid axes. The simulation with multiple size classes allows us to compare how the shape of the grains evolves, discriminating by sizes without needing to consider individual grain shapes (which would considerably increase computation time). This information cannot be obtained from the single-phase model. As the figure shows, the overall grain shape gets totally skewed towards the shape of the larger grains when a single phase is considered, and the information relative to the shape of smaller grains is completely lost. Below this graph, several ODF plots that show the difference in texture evolution for the largest and smallest grains are displayed. These differences are small, but noticeable, especially in the intensity of the rotated cube component. When a single phase is used, the ODF also resembles that of the largest grains, and the importance of these components would go unnoticed. Finally, the figure shows a 3D graph in which the relative activity in the slip systems on the 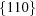 and 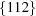 planes is represented with respect to uniaxial strain and grain size. The graph shows a large variation of slip system activity with respect to grain size from the beginning of the simulation, and a different evolution as deformation increases. Therefore, the combination of a Hall–Petch hardening law with grain-size-dependent grain shapes and textures allows for the simulation of complex behaviour that cannot be captured in more conventional simulations.

## 4. Discussion   

In this work, a new GSODF that combines the traditional grain size and orientation distributions in a single multivariate distribution function has been presented. Using a simple method, this new function can be derived from conventional experimental data. The examples in the previous section show that the GSODF has great potential in the analysis of experimental data and can be easily used to enhance computer simulations. Nevertheless, since the GSODF is a new concept, it also raises several questions that deserve to be discussed in more detail.

### 4.1. GSODF expression and derivation method   

A general expression for the GSODF has been presented in (5). Formula (9) is the specific version of (5) in which ODFs are represented using the generalized harmonic series expansion method. More specific distributions are given for the case in which the grain-size-dependent *C* coefficients exhibit a linear behaviour in (10). The GSODF may be calculated using a similar method, in principle, with any other texture representation. Other kernel functions instead of generalized harmonics could be used for the description of ODFs (several alternatives are listed in Section 1). As an extreme case, discrete textures may be used.^2^ One particularly interesting solution could be obtained in combination with orientation representation methods based on the use of quaternions as in the work of Mason & Schuh (2008 ▸). Since unitary quaternions are used to represent orientations in this formalism, additional grain size or size ratio information could be represented using non-unitary quaternions. This may facilitate the analysis and visualization of the function. Note that, since the coefficients of the generalized spherical harmonic coefficients (or *C* coefficients) and the coefficients of the hyperspherical harmonics series are linearly related (Mason & Schuh, 2009 ▸), any linear behaviour observed in the *C* coefficients will also be found when using hyperspherical harmonics.

**Figure 11:**
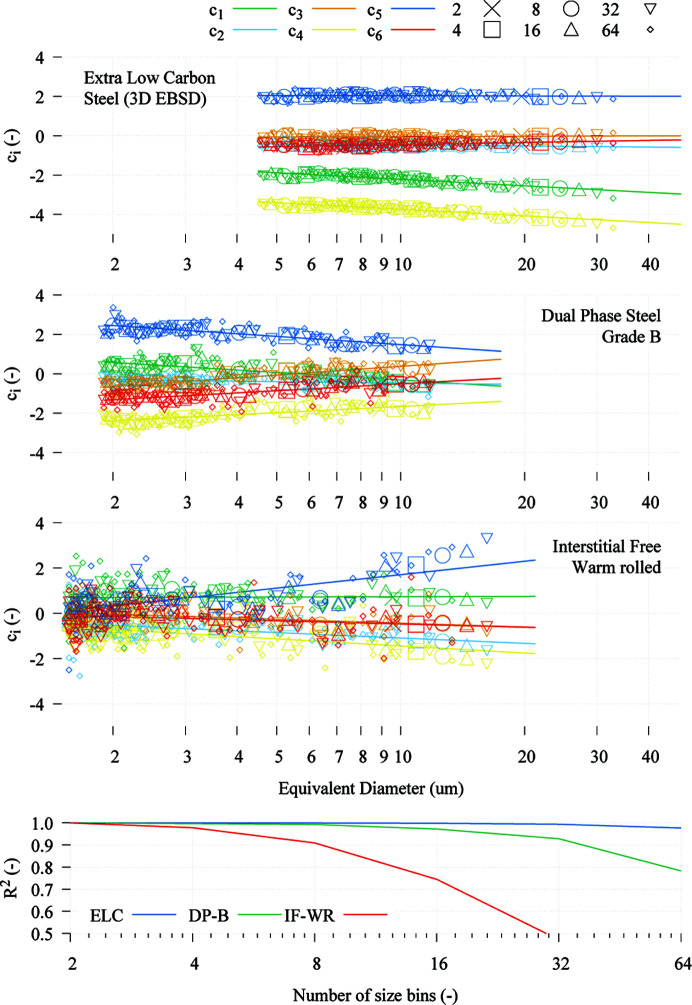
Linear fitting of *C* coefficients 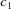 to 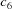 with respect to 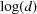 using two, four, eight, 16, 32 and 64 size bins for the ELC 3D, DP-B and IF warm-rolled microstructures. Bottom: combined 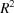 value of all the fittings with respect to number of bins.

One of the fundamental steps of the method presented here for the derivation of the GSODF is the division of the grain list into a number of size bins. In order to gain a better understanding of how the choice of this number influences the obtained results, additional fits are performed using different numbers of bins for the ELC, DP-B and IF warm-rolled materials. Fig. 11 ▸ shows the first six *C* coefficients of the ODFs calculated using two, four, eight, 16, 32 and 64 size bins. The dependence of the *C* coefficients on grain size clearly follows a linear relationship. Some divergence is observed in the results for the IF warm-rolled steel (see the 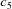 coefficient and the 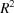 values in the bottom graph). However, the number of total grains available in this case was small (only 1127), so the number of grains used to calculate the ODFs becomes very small (less than 150 grains when eight bins are used, only 17 grains with 64 bins) and the calculated ODFs become very inaccurate.

As mentioned in Section 3.2, we did not expect to find such a simple linear expression for the *C* coefficients. This relationship has proven to also hold valid for the two DP and the two IF steel grades. Fig. 11 ▸ further reinforces this result: linearity is conserved even when the number of bins is increased. For instance, for the ELC steel, the combined 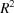 value of all the fits, each of them using a total of 64 points (bins), is almost 0.98, and it is more than 0.99 when 32 points are used. It remains to be seen if similar relationships are observed with other steel grades, metals and alloys.

**Figure 12:**
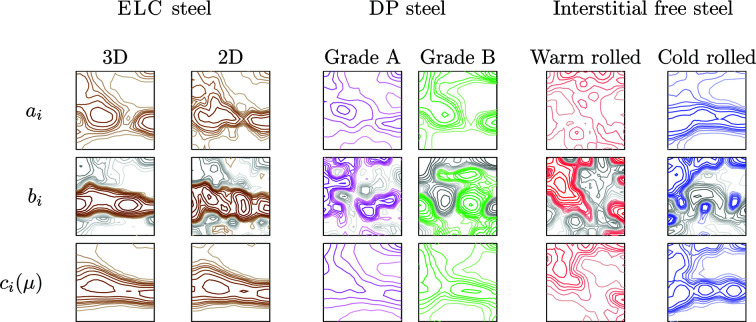
Representation of 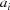, 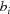 and 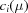 in (10) as ODFs for each of the studied cases. In the 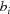 plots, negative values are displayed in grey tones. The 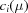 plots should correspond to the global textures in Fig. 2 ▸ (levels: see Fig. 2 ▸).

When it is known that the relationship is linear (or it is assumed to be), the fitting can be trivially performed by dividing the grains into only two size ranges. Moreover, it is a convenient method to represent grain-size-dependent ODFs in a graphical manner. The size-dependent ODF can be defined by two sets of *C* coefficients and therefore can be represented as two different ODFs: either 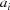 and 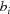 in equation (7) (two top rows in Fig. 12 ▸) or 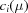 and 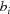 in equation (8) (two bottom rows). The combination of 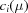 and 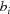 seems particularly useful: 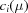 is the global ODF of the material, while 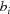 gives the variation of the ODF as the size ratios increase. Together with the grain size distribution, these two ‘ODFs’ constitute a concise visual representation of a linear GSODF.

### 4.2. Applications   

In Section 3, a number of different possible applications of the GSODF and related concepts were shown. The examples have been presented in order to offer an overview of the potential of the method and are not exhaustive. It is not difficult to envisage other problems in which it would be useful to study the correlation between grain size and crystallographic orientation. Being able to correlate texture and grain size may be useful, for example, in the study of phase transformation and recrystallization processes, where the texture for small grains could be associated with the nucleation texture and the evolution of grain-size-dependent textures with the grain growth process. Similarly, in the study of deformed microstructures, correlated orientation data could be used to better understand complex phenomena such as grain fragmentation or the formation of subgrains in substantial deformation processes. Further work is still needed to find the best way in which the concept of a GSODF can be applied to the study of multiphase materials. On the one hand, if it is possible to easily separate the existing phases, grain-size-dependent textures and misorientations could be calculated for each of these phases. On the other hand, if the distinction is challenging, the GSODF may aid in the partition of the material in different phases, taking into account possible orientation relationships between parent and product phases.

It has also been shown in Sections 3.5 and 3.6 how the GSODF can be applied in full-field and mean-field simulations. How the use of the GSODF for the generation of RVEs affects the results of full-field simulations still needs to be fully studied. Although it is expected that a more reliable representation of the microstructure will produce improved results, it is difficult to evaluate *a priori* what difference it will make. Nevertheless, even if the macroscopic mechanical response is not affected, the fact of having added microstructural information to the problem will allow additional microstructural output (*e.g.* distribution of stresses and strains over different size classes) to be obtained. Similar conclusions can be drawn from the example presented in Section 3.6. The technique presented requires further investigation, but shows potential. A similar methodology could be used with any mean-field model (also for phase transformation or recrystallization) capable of handling multiple phases in which it is meaningful to differentiate between grain size classes. This approach could also be applied in recrystallization and phase transformation models, for instance with the purpose of differentiating between nucleation and growth phenomena.

Both in full-field and in mean-field simulations, the only difference with conventional methods is that orientations are assigned as a function of grain size during the pre-processing step, but the total number of grains does not need to be increased, and the models themselves do not need to be modified in any way. Therefore, simulations can straightforwardly take advantage of the use of the GSODF to produce a more detailed (and, supposedly, more precise) output without additional costs in computation time.

### 4.3. Further possibilities   

In this work, the correlation between grain size and crystallographic orientation has proven to be a promising technique for the study of microstructures. There is no reason to think that a similar study could not be successfully performed using a different set of microstructural properties.

The technique proposed here has been extended to the study of the misorientation angle distribution in Section 3.4 and to grain shapes for the VPSC simulations in Section 3.6. A similar method may be used to find a grain-size-dependent misorientation distribution function, or to correlate a different property with orientations, such as dislocation density or intragranular misorientation. Going even further, more than two properties could be combined in a single function. A multivariate distribution function depending on *n* microstructural state variables of relevance to a particular problem (*e.g.* grain size, crystal orientation, crystal misorientation, dislocation density *etc.*) may provide a very concise description of a microstructure with much higher information density than a precise topological description.

## 5. Conclusions   

A simple method for the derivation of a multivariate microstructural distribution function including two state variables, crystal size and orientation, has been presented. The proposed distribution is expressed in the form of a single continuous function, allowing higher information density than in similar discrete representations and simplifying its analysis. Through several examples, it has been shown that studying the correlation between grain size and crystallographic orientation has the potential to aid in the analysis and modelling of polycrystalline microstructures.

For all the studied low carbon steels, a simple GSODF has been found, based on the observed linear relationship between the *C* coefficients of the harmonic series expansion of grain-size-dependent ODFs and the logarithm of the grain size. It is unknown, at this point, if a similar relationship will be found for other materials and microstructures.

It is necessary to perform further research on the correlation between grain size and orientation in other materials and after different processing routes. Moreover, the techniques presented in this work still need to be fully explored and evaluated in full studies with specific goals. Several possibilities to build on the concepts presented here have also been outlined and require more investigation. Hopefully, these concepts will form a good basis for future work.

## Figures and Tables

**Table 1:** Number of EBSD data points and grains (total and valid) for each of the materials presented in Fig. 1 ▸, and spacing between successive layers (2D EBSD scans) in 3D EBSD

Material	*Z* spacing	Points	Grains	Valid
ELC	4.0 µm	9047108	30443	28605
ELC 2D		438755	2679	2679
DP A	1.3 µm	4200420	27438	15446
DP B	1.2 µm	2691220	8527	5635
IF (warm)		259306	1376	1228
IF (cold)		5430642	16543	16247

**Table 2:** Number of points, spatial resolution (in µm) and number of grains in the three RVEs generated with *Dream3D*

Geometry	Resolution	Grains
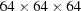	1.00	246
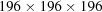	0.75	2984
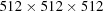	0.50	15459

## Footnotes

1 This list should not be confused with a discrete texture. In a discrete texture, orientations do not necessarily correspond to individual grains and fractions are given in relative units.

2 The combination of discrete and continuous distributions for grain size and crystallographic orientation results in four different possibilities: a continuous function, two ‘semi-discrete’ distributions (discrete in either grain sizes or orientations) and a fully discrete distribution (which would in effect be the list of grains described in Section 3.1.2).
